# Preparation of Al/Fe-Pillared Clays: Effect of the Starting Mineral

**DOI:** 10.3390/ma10121364

**Published:** 2017-11-28

**Authors:** Helir-Joseph Muñoz, Carolina Blanco, Antonio Gil, Miguel-Ángel Vicente, Luis-Alejandro Galeano

**Affiliations:** 1 Grupo de Investigación en Materiales Funcionales y Catálisis (GIMFC), Departamento de Química, Universidad de Nariño, Calle 18, Cra. 50 Campus Torobajo, 520002 Pasto, Colombia; hjmunoza@unal.edu.co; 2 Departamento de Química, Universidad Nacional de Colombia, Ciudad Universitaria, Ctra. 30 # 45-03, 111321 Bogotá, Colombia; cblancoj@unal.edu.co; 3 Departamento de Química Aplicada, Edificio de los Acebos, Universidad Pública de Navarra, Campus Arrosadia, 31006 Pamplona, Spain; andoni@unavarra.es; 4 Departamento de Química Inorgánica, Facultad de Ciencias Químicas, Universidad de Salamanca, Plaza de la Merced, s/n, 37008 Salamanca, Spain; mavicente@usal.es

**Keywords:** smectite, pillared clay, keggin-like mixed Al/Fe polyoxocation, mineralogical composition, catalytic wet peroxide oxidation

## Abstract

Four natural clays were modified with mixed polyoxocations of Al/Fe for evaluating the effect of the physicochemical properties of the starting materials (chemical composition, abundance of expandable clay phases, cationic exchange capacity and textural properties) on final physicochemical and catalytic properties of Al/Fe-PILCs. The aluminosilicate denoted C2 exhibited the highest potential as starting material in the preparation of Al/Fe-PILC catalysts, mainly due to its starting cationic exchange capacity (192 meq/100 g) and the dioctahedral nature of the smectite phase. These characteristics favored the intercalation of the mixed (Al_13−*x*_/Fe*_x_*)^7+^ Keggin-type polyoxocations, stabilizing a basal spacing of 17.4 Å and high increase of the BET surface (194 m^2^/g), mainly represented in microporous content. According to H_2_-TPR analyses, catalytic performance of the incorporated Fe in the Catalytic Wet Peroxide Oxidation (CWPO) reaction strongly depends on the level of location in mixed Al/Fe pillars. Altogether, such physicochemical characteristics promoted high performance in CWPO catalytic degradation of methyl orange in aqueous medium at very mild reaction temperatures (25.0 ± 1.0 °C) and pressure (76 kPa), achieving TOC removal of 52% and 70% of azo-dye decolourization in only 75 min of reaction under very low concentration of clay catalyst (0.05 g/L).

## 1. Introduction

Clays are products of rock erosion and are found widely distributed in nature. Their chemical and textural composition varies from one place to another, depending on their geological origin and the presence of organic and inorganic impurities [1]. Bentonite clays consist mainly of phases of the smectite group, which belong to type 2:1 materials (layers of phyllosilicates formed by two tetrahedral sheets, plus one octahedral, T:O:T) condensed in a layer, and exhibit a net neutral or negative electric structure that can vary significantly between illite, smectites, vermiculites, micas, etc. [2]. Smectites exhibit many interesting properties, especially for their application in adsorption and heterogeneous catalysis, originating in their peculiar physical, chemical and crystalline character. Among these properties, their cationic exchange capacity (CEC) and ability to swell in polar media make clays interesting as raw materials for modification through soft chemistry and intercalation methods. Pillaring has been one of the most widely used techniques in structural modification of this type of materials over the past 20 years [3,4,5].

In general, it has been documented that the success in the preparation of pillared clays strongly depends on the physical and chemical characteristics of the starting mineral. The most influential properties for this application include swellability, the net content of expandable clay minerals [4], the content of exchangeable cations as measured with the CEC, the chemical nature of such cations (in particular their radius of hydration) [6,7,8], the degree of isomorphic substitution in the tetrahedral and octahedral sheets, which indirectly is also related to the CEC, and the crystallinity of the smectite phases, among others [2,6,9,10,11,12]. The chemical composition [2] and distribution of transition metals active in the CWPO reaction, especially Fe and Cu, within the structural layers and extra-structural impurities are also important for the specific case of pillaring with the mixed Al/Fe system [13].

Al/Fe pillared clays have exhibited high performance activating the catalytic wet peroxidation in heterogeneous phase, and have therefore been used in the degradation of several organic compounds present in water, including emerging pollutants [6,14], phenolic compounds [8,15,16,17], natural organic matter [12,18] and as various toxic and bio-refractory azo-dyes [13,19,20], including methyl orange. The catalytic performance of Al/Fe-PILCs in the CWPO reaction is also closely related to the structural characteristics of the starting clay [21]. The CEC of the starting material strongly determines the ability of the Al/Fe polycations to replace the exchangeable cations originally present in the ore [8]. The amount of Fe effectively intercalated in the smectite and its specific location in the final structure of the catalyst directly affects the redox activity that the active metal may exhibit in the CWPO process, as well as its stability in the reaction medium [13]. The exchangeable cations may also play an important role as they have the ability to influence the swelling capacity of the raw material, and thus to influence the rate and the intercalation efficiency of the mixed oligomers [6]. The textural properties of the starting aluminosilicate may also be considered; the low dimensionality of the porous channels in this type of layered minerals implies that the molecules on the surface are more likely to collide with each other than they could in three dimensions. This, in turn, leads to a higher frequency of collisions and, consequently, greater reactivity [22].

Once the pillaring of the starting aluminosilicate has been carried out with the larger oligocations, the specific surface area, mainly represented in the area of micropores now available to catalyze the CWPO reaction, must substantially increase. An aspect that might negatively impact the application of a starting material in the preparation of active clay catalysts in the CWPO reaction is the presence of extra-structural Fe aggregates in the starting aluminosilicate, as these have shown to get easily leached during the catalytic action of the Al/Fe-PILCs [6]. However, in the treatment of wastewater under a continuous regime, it should not be a major obstacle. In general, the catalytic performance of these materials in the reaction-of-interest will depend on the type of iron species incorporated, its distribution, accessibility and the chemical environment of these active sites [4].

In the present work, four natural clays (denoted C1, C2, C3 and a well-known reference material BV) were, therefore, modified with a mixed intercalating ${\left(\mathrm{Al}/\mathrm{Fe}\right)}_{13}^{7+}$ solution, prepared according to a widely reported methodology [13,18,23,24,25]. The samples presented different: (i) clay content, (ii) distribution of clayey phases (iii) CEC, (iv) distribution of exchangeable cations (sodium, calcium or magnesium), (v) chemical composition, with presence of extra-structural iron aggregates, and (vi) textural properties. The physicochemical properties of the clays were evaluated before and after intercalation with the Al/Fe oligomers, and a correlation was made with the catalytic potential, exhibited by the final materials in the CWPO degradation of methyl orange in dilute aqueous solutions.

## 2. Materials and Methods

### 2.1. Materials

Four Colombian natural clays were employed as starting material, denoted as: class 1 (C1), class 2 (C2), class 3 (C3) and bentonite of the *Valle del Cauca* (BV). Clays (C1, C2 and C3) were selected and collected taking into account the following criteria: (i) they are all highly available, low-cost materials that have not been characterized or modified by pillaring procedures so far; (ii) ores from which aluminosilicates were extracted are currently exploited along with guaranteed long time exploitation horizon; (iii) besides, mineralogical analyses showed significant but different contents of expandable phases (smectite type), cationic exchange capacities and elemental composition, appropriate to figure out changes in Al/Fe-pillaring as a function of such physicochemical properties in the starting clays in the context of the heterogeneous Fenton, CWPO degradation of contaminants. On the other hand, the BV clay was used as a reference mineral as its use has been widely documented in academic literature in preparation of pillared clays [13,26,27,28]. These raw materials were refined by sedimentation of aqueous suspensions using Stokes’s law, allowing the separation of the fraction with particle diameters that are lower than 2 μm. The refined materials are then symbolized by the acronyms C1-R, C2-R, C3-R and BV-R, respectively, and were modified with the mixed intercalating solution Al/Fe prepared in diluted, standard conditions. The Keggin-type mixed oligomeric precursor was prepared using AlCl_3_·6H_2_O (99%, Sigma-Aldrich^®^, St. Louis, MO, USA), FeCl_3_·6H_2_O (97%, Sigma-Aldrich^®^, St. Louis, MO, USA) and NaOH (99%, Merck^®^, Billerica, MA, USA), used as received. Ammonium acetate (97%, Carlo Erba^®^, Barcelona, Spain) was used to determine CEC. 

### 2.2. Preparation of Pillared Clays

The source materials (C1, C2, C3 and BV) were pillared in diluted medium with the ${\left(\mathrm{Al}/\mathrm{Fe}\right)}_{13}^{7+}$ mixed system, following a standard procedure widely reported in the literature [13,18,29,30]. First, the pillaring dissolution was prepared with 0.18 mol/L of AlCl_3_·6H_2_O and 0.02 mol/L of FeCl_3_·6H_2_O solutions in an appropriate ratio to reach a final atomic ratio of 5.0% of the active metal (AMR_Fe_) in the Al/Fe solution and total metal concentration (TMC) of 0.06 mol/L after finishing the hydrolysis stage. These concentrations have proven to be optimal for the inclusion of a major fraction of the metals being part of the Keggin Al/Fe mixed polyoxocations.

**Figure 1:**
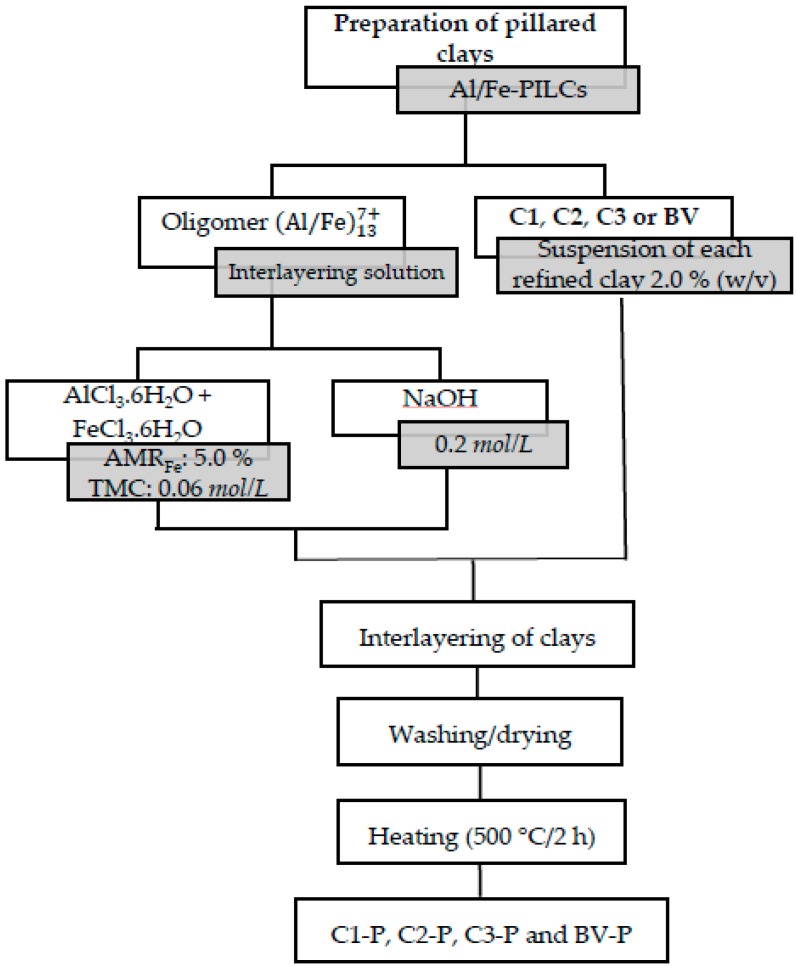
Experimental procedure of the preparation of pillared clays.

Subsequently, a 0.2 mol/L solution of NaOH was slowly added at 70 °C in sufficient quantity in order to obtain a final hydrolysis ratio (HR = HO^−^/(Al^3+^ + Fe^3+^)) of 2.4. The resulting solution was then aged at the same temperature for 2 h, and then slowly added (drop by drop) onto a 2.0% (*w*/*v*) suspension of each clay in water under vigorous stirring. Intercalated clays were repeatedly washed with distilled water using a dialysis membrane (Sigma^®^, St. Louis, MO, USA), dried at 60 °C, and calcined at 500 °C for 2 h in the open air to obtain the pillared materials (C1-P, C2-P, C3 P and BV-P). A general sketch of the experimental procedure is summarized in Figure 1. 

### 2.3. Physicochemical Characterization

Elemental analysis of the starting, extracted, and pillared aluminosilicates was performed by X-Ray Fluorescence (XRF), for which calibration curves were created using the QUANT-EXPRESS method (Fundamental Parameters) in a Bruker S8 Tiger 4 KW Wavelength Dispersive X-ray Spectrometer, with an Rh anode as an X-ray source, scintillation detector (heavy elements, from the Ti to the U), and flow (light elements, from the Na to the Sc). For this analysis, approximately 1.0 g of sample was used, sieved through a 400 mesh, and calcined at a heating rate of 3.0 °C/min up to 950 °C in order to determine the ignition losses.

The CEC of the refined and pillared clays was determined by saturation at reflux temperature using 45 mL of 2.0 mol/L ammonium acetate solution per g of solid, followed by repeated washing with distilled water and centrifugation to remove excess of ammonium ions. The content of exchanged ${\mathrm{NH}}_{4}^{+}$ ions was then determined by the micro-Kjeldahl method and finally expressed as meq. ${\mathrm{NH}}_{4}^{+}$/100 g of solid [16].

X-ray diffraction (XRD) patterns of the raw, refined (starting materials), and pillared minerals were determined using a Bruker D8 Advance diffractometer, operating at 40 kV and 30 mA with a scanning speed of 2.29 °2θ/min, employing Cu Kα radiation (λ = 0.15416 nm). The materials were analyzed by default in the range of 2.0° to 70.0°. Determinations on oriented specimens were measured in samples deposited on glass plates and dried at room temperature in a range between 2.0° and 30°.

For the semi-quantitative determination of the smectite content in every mineral, the methodology proposed by Thorez [31] was adopted, measuring the diffractograms of oriented plates of the initial, raw aluminosilicates (C1, C2, C3 and BV), consecutively performing the expansions on each specimen with ethylene glycol and finally thermal collapse of the aluminosilicate layers (T = 400 °C/2 h). The preparation of the oriented plates was performed in the following manner: first, an extensive cationic exchange of each of the materials was carried out using a 0.5 mol/L CaCl_2_ solution. The suspensions of each Ca^2+^-homoionized mineral were well dispersed with ultrasound for 15 min, and then deposited with a Pasteur pipette on small glass sheets fitting the sample holder of the diffractometer. They were then allowed to dry at room temperature and the diffractograms of the oriented films measured. Afterwards, the plates were saturated with ethylene glycol, according to the methodology proposed by Moore et al. [32], in which the plates were solvated by exposing them to solvent vapor in a desiccator at room temperature for 16 h. A few minutes after the treatment’s finish, the plates were removed and the diffractograms measured again under the same conditions. Finally, the same specimens were calcined for 2 h at 400 °C and measured again by X-ray diffraction.

The abundance of each clay phase in the minerals was obtained from the integrated areas under the *d*_001_ signal for smectite, illite, dividing each value by an empirical factor established as 1 for illite, 4 for smectite [30]. The textural analysis of the solids was carried out by determining the nitrogen adsorption isotherm at −196 °C, obtained from a 100–200 mg sample in a 3-Flex Micromeritics Sorptometer, over a wide range of relative pressures, previously the samples degassed at 300 °C for 12 h. The BET specific surface areas (S_BET_) were determined by the multipoint model, using Keii-Rouquerol criteria to find the best linear BET fitting [1]. The external surface (S_ext_) and the surface corresponding to micropores (S_μp_) were calculated using the *t-plot* model. The micropore size distributions were calculated using the method of Horvath and Kawazoe, suitable for the morphology of porous slit type predominant in pillared clays [30].

Hydrogen temperature-programmed reduction analyses (H_2_-TPR) were performed using a Micromeritics TPR/TPD 2900 apparatus. About 40 mg sample was heated from room temperature to 900 °C at 10 °C/min under a flow of 60 mL/min of reactive gas (5.0% H_2_ in Ar, Air Liquide, Madrid, Spain). Hydrogen consumption was measured with a thermal conductivity detector (TCD), where CuO (Merck 99.99%) was used as the external calibration standard. Based on the measured thermal events, Fe reduction signals were differentiated in terms of the sites present in Al/Fe-PILCs as proposed in advance [13]: fraction of extra-structural FeOx aggregates, fraction of interlayered iron oxide aggregates (iron oxides “decorating” alumina pillars), Fe occupying structural sites of the clay, and finally the Fe forming part of real mixed pillars Al/Fe.

### 2.4. Catalytic Experiments

The modified materials (C1-P, C2-P, C3-P and BV-P) were evaluated as active solids in hydrogen peroxide-assisted catalytic oxidation of methyl orange (MO). The experiments were carried out at 25.0 ± 1.0 °C and atmospheric pressure (76 kPa) in a 1500 mL Semibatch (Pyrex^®^, New York, NY, USA) glass reactor equipped with a jacket for temperature control with thermostatic bath and a peristaltic pump to feed the H_2_O_2_ solution under controlled flow. For each test, the reactor was loaded with 1.0 L of MO solution (Sigma Aldrich, St. Louis, MO, USA, 85%) 0.119 mmol/L and 0.5 g of the solid catalyst to be evaluated (0.05 g of catalyst/L), under constant both air bubbling (about 2 L/min) and mechanical stirring (600 rpm). Addition of 100 mL of the H_2_O_2_ solution (Panreac, Barcelona, Spain 50%) 51.19 mmol/L (equivalent to exactly the stoichiometric theoretical amount for full mineralization of the azo-dye in the reactor) started after 15 min of stirring and air bubbling (equilibrium period) at flowrate of 2.0 mL/min. The zero time of reaction was the starting point for the addition of the hydrogen peroxide solution into the reactor; from that moment on, 25 mL samples were taken, during a total reaction time of 1 h. The samples were micro-filtered (Millipore, Burlington, MA, USA, 0.45 μm filters) to separate the catalyst previous to analysis. The pH of the solution was adjusted to 3.4 and constantly controlled in this value through the addition of drops of 0.1 mol/L HCl or NaOH [13]. 

## 3. Results and Discussion

### 3.1. Physicochemical Characterization

The chemical compositions of the source minerals (C1, C2, C3 and BV) are shown in Table 1. The SiO_2_/Al_2_O_3_ ratios of materials C2, C3 and BV were much closer to three, indicating the possible presence of smectites [33,34]. Material C1, presented a SiO_2_/Al_2_O_3_ ratio higher than 3.5 and a high content of potassium, which may suggest that this aluminosilicate is mainly constituted of illite. It indirectly shows that C1 presents an important content of collapsed layered-clay phases, since potassium usually lodges strongly in the ditrigonal holes of the tetrahedral layers of the aluminosilicate, hindering any subsequent ion exchange process [31,32]. The presence of iron in all raw minerals (from 7.86 to 19.94% *w*/*w*) (Table 1) could be explained because this metal can serve as an isomorphic substitute for both Si and Al within the layered structure. In addition, impurity phases can also be found on the surface of the sheets of the material in the form of extra-structural oxides. This element was present in a higher percentage in the aluminosilicate C3 (19.94% *w*/*w*), which may indicate that this mineral exhibits large amount of extra-structural impurities of iron, or that the metal is part of the layers of the material (due to the high content of structural Fe) whose smectitic phase could correspond to nontronite or ferruginous [35]. 

Regarding the magnesium content, C2 was found to have a lower content (2.64% *w*/*w*) compared to BV (3.16% *w*/*w*) and C3 (3.08% *w*/*w*) (see Table 1), indicating that the smectite phase that may be present in this material would be mostly dioctahedral, whereas BV and C3 could be trioctahedral phyllosilicates. The Na and Ca contents in the starting phyllosilicates are correlated with the exchangeable cations present in their interlayer space. According to the results, the materials with the highest sodium content are C1 (3.28% *w*/*w*) and C3 (3.65% *w*/*w*), whereas the phyllosilicates C2 and BV have the lowest sodium content (1.55 and 0.74% *w*/*w*, respectively), as well as calcium (2.19 and 0.93% *w*/*w*, respectively). These characteristics are important if one takes into account that when the interlaminar cation is sodium, the smectites have a greater capacity of swelling in polar solvents. It may conduce to complete dissociation of individual smectite crystals, resulting in a higher degree of dispersion and maximum development of colloidal properties. If Ca or Mg are the predominant exchange cations, their swelling and cationic exchange capacities are much lower [6,21]. 

The SiO_2_/Al_2_O_3_ ratio also provides information on the amount of quartz that may be present in the starting clays [36]. A high SiO_2_/Al_2_O_3_ ratio indicates a greater amount of quartz in the raw material. Thus, according to Table 1, C1 showed the highest SiO_2_/Al_2_O_3_ ratio with a value of 3.65, followed by C2 (3.22), C3 (2.93), and finally BV (2.60). However, after purification, the SiO_2_/Al_2_O_3_ ratio of these materials decreased as a result of the significant removal of the quartz content; clearly, the most significant decrease was from material C1, suggesting it was the mineral most affected by quartz impurity. In the case of C3, the SiO_2_ content indeed became slightly higher after purification, showing that this mineral was the least affected by quartz contamination, but also probably because the loss of iron oxides was more significant. 

**Figure 2:**
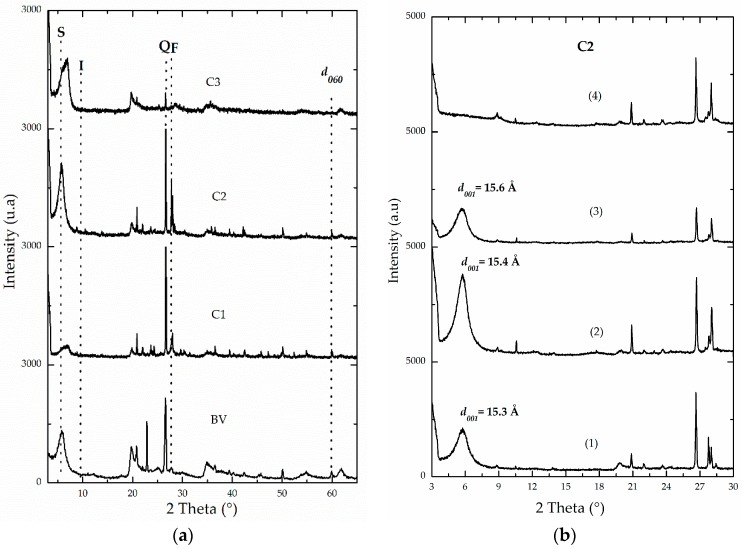
X-ray diffraction patterns of: (**a**) powders of untreated BV, C1, C2 and C3 raw minerals. (S: smectite, I: illite, Q: quartz, F: Feldspars); (**b**) oriented films of C2 clay under following conditions: (1) untreated, (2) Ca^2+^-homoionized, (3) saturated with ethylene glycol and (4) calcined at 400 °C/2 h.

Semi-quantitative mineralogical analyses were carried out using XRD on monoaxial-oriented films. This mode makes use of the spontaneous stacking property of the laminar clays depending on drying conditions, and allows for films to be arranged in a parallel fashion when set to dry from suspensions diluted at room temperature [31,32]. In layered materials, it is more advantageous than determinations on powdered samples, which given the reduced size of the clay particles, implies that the coherent domain in a single crystal will not give important diffraction maxima and the peaks or signals will become invisible or slightly visible. As XRD quantitative analysis of clay minerals is a very complex task, it should be noted that the one adopted here is a semi-quantitative determination. One of the most complicated variables to control in quantitative approach is the selection of a standard mineral whose diffraction characteristics should be identical to those of every crystalline phase present in the ore-of-interest. Another drawback is that the intrinsic intensity of diffraction of a given mineral also depends on its chemical composition, which is clearly quite variable in this type of natural sample. Powder diffractograms (Figure 2a) of all starting aluminosilicates revealed the presence of illite (I), quartz (Q) and, in the material C2, a very intense signal was observed corresponding to the feldspars (F) present as impurity in the material. Hence, the semi-quantitative estimation of the mineralogical composition of clay phases present in the raw minerals (Table 2), determined that BV and C3 were the materials with the highest contents of smectite (S), followed by material C2 (60%, 54% and 46%, respectively). Meanwhile, material C1 was basically constituted of non-expandable clay minerals, specifically illite-type (I), and is strongly contaminated by quartz as shown before [37]. These results correlate very well with what was discussed concerning elemental analysis. Then, C1 could be expected to exhibit lower expandability than the rest of the raw materials when intercalated and pillared with the mixed oligomeric Al/Fe system [2,4,21]. In Figure 2b, it is shown that the relative intensity and definition of the reflection corresponding to the basal spacing (001) in smectites significantly increased in the aluminosilicate C2 after carrying out an extensive exchange process with the divalent cation Ca^2+^, followed by the process of saturation with ethylene glycol in comparison to the raw starting mineral. The same general behavior was observed in the rest of the materials (Supplementary Material, Figure S1). In addition, this reflection in the saturated C2 material slightly shifted to lower 2° theta angle, evidencing the swelling capacity of the smectite present in this aluminosilicate [4]. The smectite phase appears to be well crystallized as it exhibits a peak of strong and acute intensity close to 15.0 Å (Figure 2b), revealing that the predominant exchangeable cation in this aluminosilicate could be calcium and/or probably also magnesium, as suggested by XRF analyses (see Table 1). The signals of the non-expandable phases, commonly found accompanying this type of naturally occurring aluminosilicates, clearly decreased in relative intensity (not shown) as the refining by sedimentation process was carried out; in addition, it generated a remarkable increase in the relative intensity of the signal corresponding to basal spacing in refined materials, especially in BV-R and C2-R solids. It was also reflected in the XRF results (Table 1); as discussed above, both materials showed a decrease in SiO_2_ content by about 3.0% with respect to their starting raw minerals (BV and C2).

An important increase in the CEC (see Table 3) was observed in the refined materials compared to the raw ones; from C1 to C1-R (40 meq/100 g), from C2 to C2-R (74 meq/100 g) and from BV to BV-R (13 meq/100 g) as a product of the elimination of non-expandable phases such as quartz. However, C3 was the only material that did not show an increase in CEC, but rather a decrease upon refinement (C3-R 10 meq/100 g). It may suggest that at least a part of the content of Fe (19.94% *w*/*w*, Table 1) in this material, possibly as an impurity, was exchangeable and contributed to the CEC of this mineral, but obviously less in the case of its refined form.

The *d*_060_ signal of C1 material showed the trioctahedral nature of its clay phase (59.9°; d = 1.542 Å), whereas dioctahedral for the rest of materials: 62.0° (d = 1.50 Å) for C2, 61.6° (d = 1.504 Å) for C3 and 61.9° (d = 1.504 Å) for BV (Figure 2a). According to several authors, the range of values for this spacing typical in dioctahedral smectites is 1.49 and 1.52 Å, and it oscillates between 1.52 and 1.54 Å for trioctahedral phases [31,32]. 

The textural properties of the raw materials (Table 3) showed that the one exhibiting the higher S_BET_ was C3 (113 m^2^/g); it could be related to its higher Fe content, whose extra-structural fraction may correspond to iron oxides. The extra-structural Fe oxides generally display high external surfaces [38]. In the materials C2 and BV the external surfaces were higher (47 m^2^/g and 49 m^2^/g) than that of C1 (30 m^2^/g), which probably relates to the higher content of clay-like phases present in both materials, whose particle sizes are characterized to be smaller than 2 μm. This is due to the refining step, as non-expandable phases such as quartz were retired, resulting in an increased abundance of clay phases in the refined solids, as well as an increase of the S_Ext_ with respect to the starting minerals, as it can be observed in Table 3. The increase in the specific surface area of the refined material BV-R (97 m^2^/g) with respect to its starting raw aluminosilicate (BV, 85 m^2^/g) was lower in contrast to the increase observed in C1-R and C2-R refined clays; it correlates with the higher content of smectite present in this material. The refined clay C1-R was the material showing the largest increase in the specific surface area (up to 105 m^2^/g) with respect to its crude aluminosilicate (45 m^2^/g), which from the point of view of the mineralogical composition is somehow expectable, since it had a higher level of impurities, as discussed above. The starting mineral C3 was the only one whose textural properties did not get enhanced upon refining by particle size; it correlates very well with the above-explained high content of iron oxides displayed by this sample. It is noteworthy that the specific BET surface (S_BET_) of all the starting raw minerals was mostly represented in external surface, related with the smallest average particle size of the clay fractions, together with their low accessibility into the microporous region (interlayer zone). 

As a result of the modification of the starting materials with the mixed Al/Fe oligocationic system (C1-P, C2-P, C3-P and BV-P), all products exhibited an increase in content of both metals (Table 1). However, it should be noted that the C2 material exhibited the highest efficiency incorporating iron (2.37% *w*/*w* as Fe_2_O_3_) and Al (8.89% *w*/*w* as Al_2_O_3_), which happened together with the highest fraction of compensated CEC in the series of minerals (59%; Table 3). It clearly demonstrates that Al/Fe mixed oxides got preferentially stabilized in the interlayer space of the clay, following the targeted cationic exchange mechanism (Table 1). These results are in accordance with those obtained on the widely reported BV-P, reference aluminosilicate [18,26,39]. It got also corroborated as the C2-P pillared material exhibited a decrease of about 90% in its content of the exchangeable cation (Ca^2+^) relative to its starting, refined material (C2-R) (Table 1). The other materials modified with the mixed Al/Fe system (BV-P, C1-P and C3-P) also showed a decrease in their contents of exchangeable cations (Na^+^ and Ca^2+^) with respect to their starting materials, suggesting an exchange and fixation of the intercalating metals in the interlayer space, but not too preferentially as seen for C2-P. 

**Figure 3:**
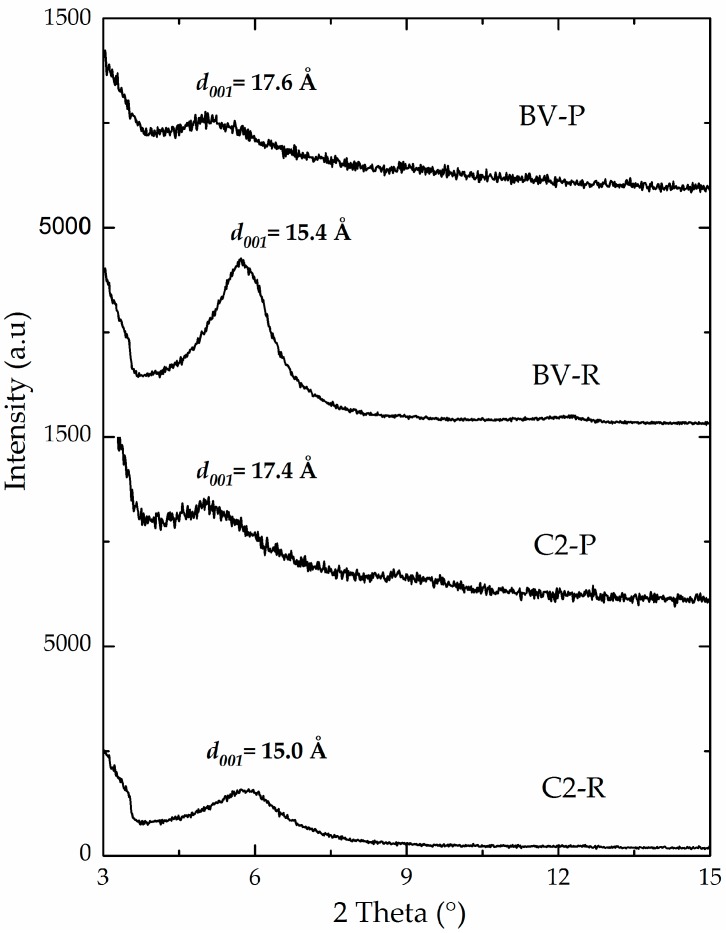
X-ray powder diffraction patterns of refined and pillared materials.

The intercalation of the Keggin-type polyoxocations in C2-P and BV-P materials was also evidenced by the expansion of the basal spacing of these aluminosilicates. It is clearly illustrated through the slight shift of the *d*_001_ signal to lower 2 theta angles (Figure 3), compared to their corresponding starting, refined materials (C2-R and BV-R). Final basal spacings of both materials were 17.4 Å and 17.6 Å, respectively. These values were only slightly lower than the reported statistical diameter of the Keggin polycation (about 8.9 Å) [2,40,41], which is expected to decrease by heating at high temperatures as in this case. It should be noted that C1-P material exhibited the lowest final basal spacing (16.9 Å, Table 3), probably due to its lower content of expandable phases, therefore leading to lower capacity to intercalate Keggin-type Al/Fe polyoxocations in its interlayer space.

As a result of the pillaring process, the starting clays increased their BET-specific surface areas by approximately 102, 132, 77 and 54 m^2^/g for C1-P, C2-P, C3-P and BV-P, respectively (Table 3). Therefore, it can be easily observed that such increase in S_BET_ was predominantly due to formation of microporous surface (S_μp_). In addition, it can be inferred that higher textural properties obtained on final pillared material correlated to a great extent with the nature of the starting material previously exhibiting higher CEC, cationic compensation and efficient incorporation of Fe and Al. Therefore, it can be stated that, in general, the CEC of the starting mineral is a fundamental parameter in order to anticipate successful modification via intercalation-pillaring. In this regard, C2 material certainly presented the highest final specific surface area upon Al/Fe-pillaring (C2-P) (194 m^2^/g), indicating that it was the aluminosilicate that developed the greatest expansion. C3-P also showed high final specific surface area (185 m^2^/g, Table 3) quite comparable to C2-P. However, its external area was larger than the one found in pillared material C2. It means that even after the pillaring step, an important fraction of Fe oxide aggregates persisted on the external surface of the particles, which could be disadvantageous from the catalytic point of view. This type of iron content has been shown in preceding studies to be more easily leached in the CWPO strongly oxidizing environment featuring this reaction [13]. 

**Figure 4:**
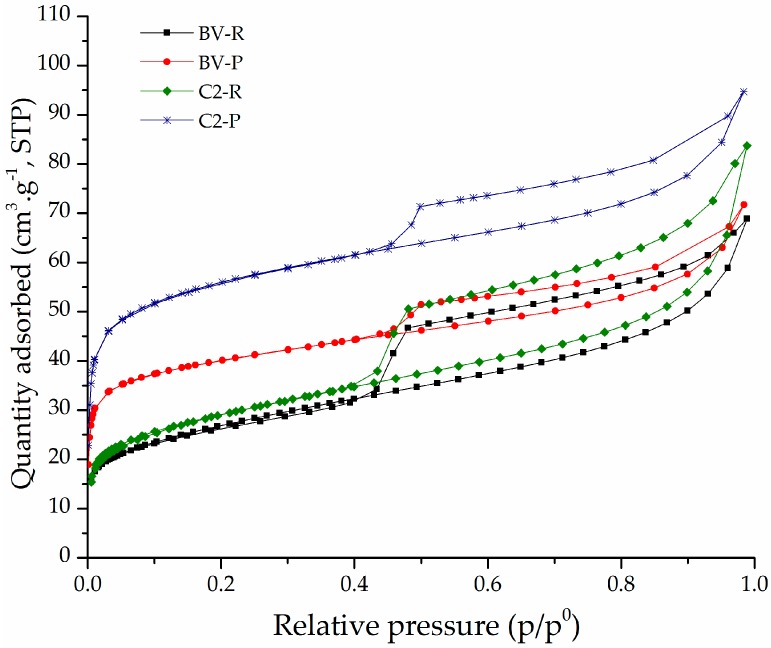
Horvath and Kawazoe micropore width distributions of refined (R) and pillared (P) forms of aluminosilicates C2 and BV.

The adsorption–desorption isotherms of the refined and pillared clays are shown in Figure 4. The isotherms of the starting refined materials are type IV (a) according to the IUPAC system (Figure 4 and Figure S2 in Supplementary Material) [42], corresponding to mesoporous materials [43]. The C3-R aluminosilicate presented an adsorption–desorption curve with a higher separation between the branches of adsorption and desorption; it means this material presented high capillary condensation [4]. The isotherms of the pillared aluminosilicates were intermediates between type I, in the range of low relative pressures, and IV, at high p/p^0^, according to BDDT classification [44], indicating the presence of both micropores and mesopores. These solids exhibited H3 hysteresis in the IUPAC classification [42], a characteristic behavior of lamellar aggregates of non-homogeneous size and/or shape, whose particles form flexible pores with slit-like morphology [4,45], typical in pillared clays. 

**Figure 5:**
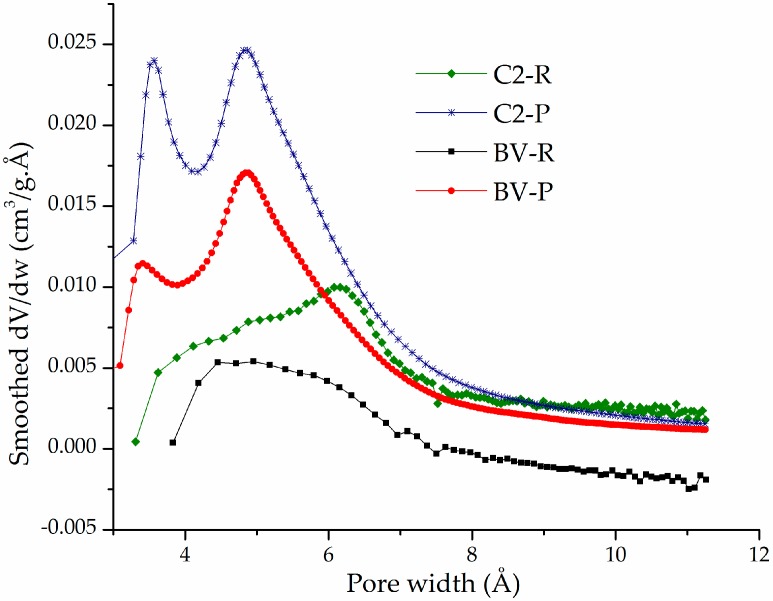
Horvath and Kawazoe micropore width distributions of refined (R) and pillared (P) forms of aluminosilicates C2 and BV.

The Horvath–Kawazoe (HK) distributions of micropore widths (Figure 5) of the pillared materials C2-P and BV-P showed well-defined bimodal behavior: (*i*) a mean pore width around 3.5 Å, which probably corresponds to a fraction of the aluminosilicate interlayered by small, poorly oligomerized Al/Fe species in the intercalating solutions and (*ii*) average pore width centered at 5.0 Å that could be ascribed to the second fraction interlayered with those formerly condensed Keggin-like oligocations, whose statistical diameter (8.9 Å) is obviously expected to decrease after the period of heating at high temperature. Such a last dimension apparently corresponds in relatively poor accord to the expected dimensions for the pores provided by the XRD-measured basal spacings in these two materials (17.4–17.6 Å), after subtraction of the layer thickness featuring the TOT aluminosilicates (around 9.96 Å). However, it is expected to significantly decrease after treatment at high temperature but, in addition, it must be considered that measurements with nitrogen as adsorbate within such a range of very low pressures, corresponding to pore widths below 10 Å, are well-known to deviate in around 2.0 Å towards lower dimensions than actual because of the Van der Waals interactions of nitrogen with such narrow pore walls. It means, the average pore widths truly found in pillared materials C2-P and BV-P were indeed close to 5.5 Å and 7.0 Å, respectively, in higher level of fitting against the theoretical dimensions of the interlayered mixed Keggin-like oligocations. It is noteworthy that a clear difference is observed among the diagrams generated for the pillared forms against those corresponding to the starting, refined forms of both aluminosilicates (C2-R, BV-R); it is obvious that micropore contents measured in the pillared materials were practically absent in the starting, refined materials, whose broad distributions of pore widths were not significantly below 8.0 Å. 

**Figure 6:**
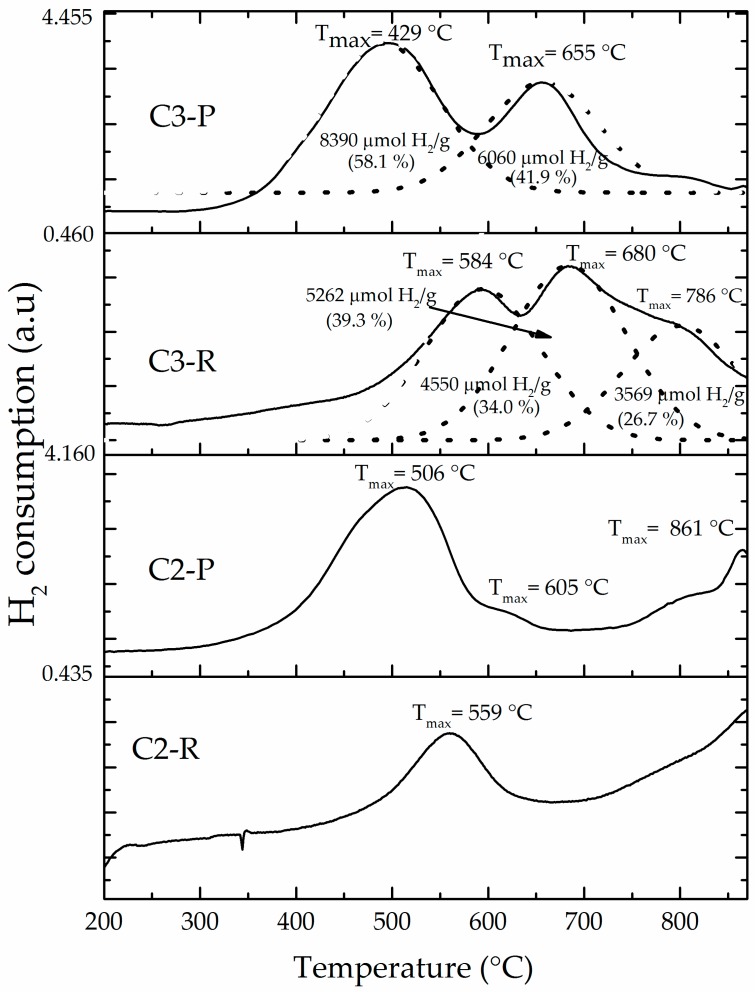
Hydrogen–temperature programmed reduction (H_2_–TPR) of refined (R) and pillared forms of clays C2 and C3: Solid lines are experimental curves and dotted lines Gaussian fitted curves of C3-derived materials.

The H_2_-TPR profile (Figure 6) of the starting aluminosilicate C2-R (4307 μmol H_2_/g) showed two reduction events centered at around 560 °C and slightly over 800 °C. These can be attributed primarily to the reduction of extra-structural iron (iron oxides, external polluting phases), and secondly to less-reducible structural iron species being part of the aluminosilicate sheets. Meanwhile, C3-R showed three broader, overlapped events of reduction at slightly higher temperatures (450–870 °C) (the curves were fitted into three peaks by the Gaussian method), whose assignments are similar, with the extra signal (peak to 786 °C) at higher temperature probably suggesting the very high Fe content present in this sample (XRF, see Table 1) to be also distributed in various structural sites within the aluminosilicate structural framework (e.g., Fe occupying both octahedral and not only tetrahedral structural holes in the clay layers). A high total hydrogen consumption (13,381 μmol H_2_/g) was of course also verified for this material C3-R. The peak deconvolution for diagrams of C3-R and C3-P materials shows, as expected, a slightly increased total consumption of hydrogen displayed by C3-P against C3-R (see Fe_2_O_3_ contents, Table 1). In addition, hydrogen consumption of peak 1 in C3-P does not exceed the sum of the first two peaks in thermogram of C3-R. Peak 2 consumption (C3-P) does not exceed sum of peaks 2 and 3 in C3-R. It may suggest that the first peak in C3-R got fully anticipated in temperature in C3-P, but the second peak only partially and then distributed between peaks 1 and 2 in C3-P. Finally, the third peak (broad shoulder) in C3-R got fully anticipated in C3-P second peak. But more interestingly, it is evidently obvious that iron incorporated in C3-P material as a product of the pillaring procedure got widely distributed including extra-structural oxides, being reduced at lower temperature, together with interlayered forms more probably reduced in the second peak of the C3-P diagram. It must be stressed that peak deconvolution of C3-P signal did not consider final consumption (over 680 °C) as an extra peak and then, this consumption is included in the 41.9% displayed in the second peak of the C3-P material. Therefore, hydrogen consumption due to iron interlayered in “truly” mixed Al/Fe-pillars would correspond to such a reduction taking place at the highest temperature. 

The reduction profiles of all the modified materials (Figure 6 and Figure S3, supplementary material) clearly showed that the first thermal event anticipates its reduction temperature as a result of the pillaring procedure, probably due to increased access to the active sites, making them more easily reduced [16]. However, it should be noted that BV-P and C2-P materials exhibited very similar reduction profiles (Supplementary Material, Figure S3), in which only one instead of two thermal events of reduction was observed at approximately 500 °C corresponding to the aforementioned external oxides. According to bibliographic reports [20], it could be ascribed to FeOOH deposited on the external surface of the clay mineral. This is evidence of the effective pillaring of the C2 aluminosilicate, since the BV-P material, widely reported before, could be used as a reference [45]. In addition, the H_2_-TPR diagrams of these materials also showed important hydrogen consumption approaching 900 °C, in good agreement with what was observed elsewhere before [13] for the system Al/Fe-PILC obtained from the same mineral BV, where it was attributed to truly mixed Al/Fe-pillars taking place in the structure. The C2-P material exhibited a shoulder close to 605 °C that, according to earlier interpretation, might correspond to the reduction effect of iron oxide aggregates under strong interaction with Al_2_O_3_ or the so-called iron “decorating” the alumina pillars [13]. This shoulder may appear overlapped in the C3-P material due to its high content of extra- and structural iron. Besides, this material showed a decrease in the full consumption of hydrogen (12,271 μmol H_2_/g) in comparison with its starting material (13,381 μmol H_2_/g); it might be due to leaching of a fraction of the extra-structural iron present in the mineral during the pillaring procedure, in good correlation with what was observed in the elemental analyses (Table 1). 

### 3.2. Catalytic Performance of the Al/Fe-PILCs

**Figure 7:**
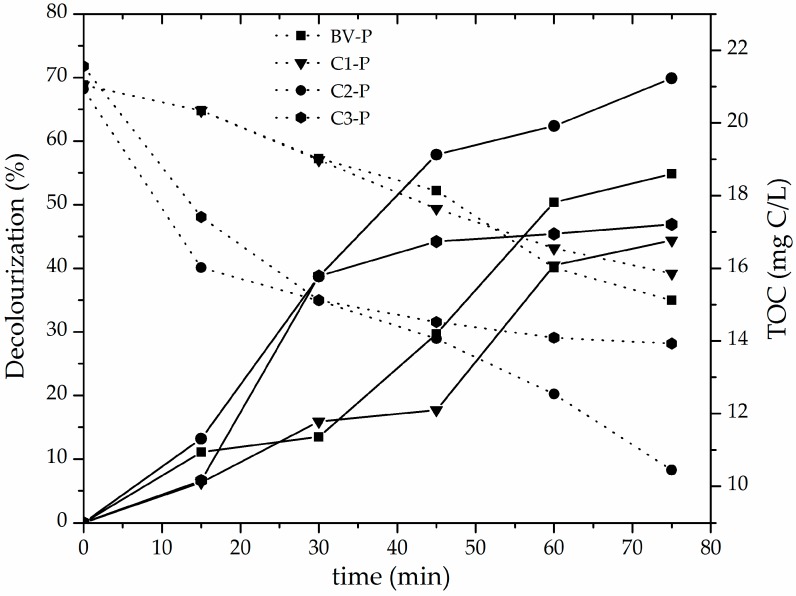
Catalytic behavior of pillared clays in terms of methyl orange decolourization (solid lines) and TOC concentration (dotted lines): Catalyst loading = 0.05 g/L; [MO]_0_ = 0.119 mmol/L; [H_2_O_2_]_added_ = 51.2 mmol/L, V_added_ = 100 mL; H_2_O_2_ stepwise addition = 100 mL/h; pH = 3.4; T = 25 ± 0.1 °C; ambient pressure = 0.76 atm.

The evolution of decolourization (%) and total organic carbon concentration in the methyl orange solutions throughout the CWPO catalytic reaction using the set of pillared clays as active materials are compared in Figure 7. It can be clearly seen that the C2-P catalyst exhibited the highest catalytic efficiency as a function of both parameters (decolourization about 70%; final TOC content about 10 mg/L, equivalent to around 52.2% of TOC degradation) in only 75 min of reaction at very mild conditions of either temperature (25 °C) of reaction and pH (3.4). These results mainly correlate with the following physicochemical properties of the pillared materials, in this order: (i) the higher amount of incorporated Fe (Table 3), with a high fraction of the transition metal being part of truly mixed Al/Fe pillars according to H_2_-TPR diagrams (Figure 6); (ii) the high reached final specific BET surface area (mainly represented as microporous content); (iii) the high starting CEC and its fraction that finally resulted compensated by intercalating polycations (% CC in Table 3). Such a set of physicochemical properties in starting aluminosilicates apparently govern in higher extent the final reactivity of the pillared materials promoting the CWPO catalytic activation of hydrogen peroxide (e.g., internal diffusion of the oxidizing agent towards the active sites present in the catalyst, thus generating a greater efficiency in the production of hydroxyl radicals). In spite of the widely spread criteria around this topic, best performing pillared material did not display higher basal spacing, which suggests that this parameter may vary more widely as a function of the experimental procedure in preparation. Moreover, it was not too different in comparison to the rest of materials studied here or other typical values already reported for Al/Fe-PILCs [4,13]. In addition, it is worth mentioning that previous particle size refining significantly affects the final success of the pillaring process; for instance, C2 material showed only the third higher CEC in raw minerals, but the first in refined forms. It must be stressed that C2-P aluminosilicate presented better catalytic behavior than the BV-P material widely used and reported before in the scientific literature relevant to the field [13,26,27], displaying catalytic performance comparable to those reported in the literature for the CWPO reaction of either methyl orange or orange II in terms of decolourization [13,39,46] using modified clays with the following metal systems: mixed Al/Fe ~80% (75 min of reaction) or MnS ~70% (4 h of reaction). It must be stressed that maximal decolourization reported here was obtained at only 45 min of reaction (plus 30 min of pre-equilibrium period) compared to more than 3 h of reaction [13] or 30 min of reaction (plus 15 min of pre-equillibrium) [39], respectively, reported before. Meanwhile, TOC removal exhibited by Fe-impregnated saponite in decolourization of Orange II azo dye was around 60% (3 h of reaction) [46]. However, it is noteworthy that in some of such cases along with longer times of reaction, higher catalyst loadings were used as compared with this study. Of course, it may display even better maximal values of azo-dye decolourization under optimal conditions of catalyst and peroxide concentrations; it should be noted that a low concentration of catalyst was employed in this study in order to get the highest possible difference in the responses among tested materials. Regarding the effect of the presence of phase impurities such as illite, quartz and feldspar (collapsed or difficult to swell phases) in the starting clay identified in this research, it may influence the physicochemical properties of the starting materials such as the CEC. This means the ability of the aluminosilicate to undergo subsequent ion-exchange processes of the Keggin-like Al/Fe-polyoxocations into interlayer space; of course, it might also indirectly influence the efficiency of incorporation of the active phase, the expansion level of the material and, in turn, final textural properties. However, as far as we know, such a type of impurities have not shown direct influence on the catalytic behavior of the materials (for instance, by transition metals located at structural sites of the phases catalyzing the reaction). In spite of very recent studies claiming the CWPO catalytic activity displayed by naturally occurring minerals [47], they have been mostly iron-rich minerals (namely, hematite, magnetite and ilmenite) exhibiting in addition significant Fe-leaching, pretty longer times and higher temperatures of reaction than those reported here.

**Figure 8:**
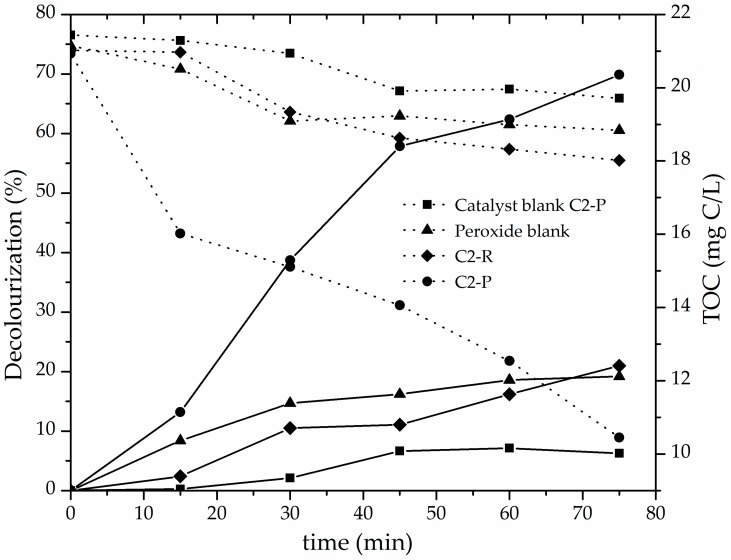
Decolourization (solid lines) and TOC concentration (dotted lines) shown by refined and pillared forms of C2 material in the CWPO degradation of methyl orange: Catalyst loading = 0.05 g/L; [MO]_0_ = 0.119 mmol/L; [H_2_O_2_]_added_ = 51.2 mmol/L, V_added_ = 100 mL; H_2_O_2_ stepwise addition = 100 mL/h; pH = 3.4; T = 25 ± 0.1 °C; ambient pressure = 0.76 atm.

Finally, it can be seen in Figure 8 that catalytic performance of C2-P material could not be ascribed to either catalytic response of the Fe content already present in the starting refined mineral, simple adsorption on the surface of the pillared clay or straightforward oxidation by the molecular, non-activated H_2_O_2_. C2-R material barely reached 20% decolourization and 14.3% of TOC degradation, C2-P material blank (no peroxide added) 6.2% decolourization and 8.0% of TOC degradation, whereas the catalytic system only in the presence of peroxide with no solid catalyst added (peroxide blank) reached less than 20% of bleaching and mineralized only 10.9% of the TOC initial content. It means that the catalytic activity displayed by the C2-P solid was mainly due to the degradation of the azo-dye contaminant by hydroxyl and other radicals generated on the active sites of the modified material, and not to simple adsorption, nor direct attack of the oxidizing agent on the contaminant molecules regarding the effect of the mineralogical composition.

## 4. Conclusions

In this work, the effects of starting mineral on the physicochemical and catalytic properties of the mixed Al/Fe-pillared clays were studied. It was shown that pillaring procedure on C2-R (starting, previously refined aluminosilicate) favored the best response in the catalytic wet peroxide oxidation of the azo-dye methyl orange in aqueous solution. The highest catalytic performance of this material correlated mainly with following properties of their raw starting and/or refined forms, in this order: the high amount of incorporated Fe with a significant fraction of it being part of truly mixed Al/Fe pillars, high specific BET surface area (mainly represented as microporous content), high starting CEC and its fraction finally compensated by intercalating polycations and basal spacing close to 18 Å. In general, dioctahedral smectites showed better performance in structural modification and catalytic application. In spite of the widely accepted criteria, best performing pillared material did not display higher basal spacing within the study. It suggested that this parameter may closely vary around the targeted space featuring Keggin-like pillared clays, but the textural and catalytic properties deviate seriously as a function of other experimental factors. Low-pressure adsorption isotherms of N_2_ treated by the Horvath–Kawazoe (HK) model revealed, for the first time, a bimodal distribution of micropore widths taking place in Al/Fe-PILCs. In summary, pillared aluminosilicate (C2-P) exhibited the best catalytic performance in the CWPO catalytic reaction of methyl orange degradation. This material achieved up to 70% of decolourization and 52% of mineralization of the starting TOC present in solution, under soft reaction conditions (RT, 25 °C, 0.05 g/L of clay catalyst and stoichiometric, no excess of H_2_O_2_). It was proved that the catalytic performance exhibited by the material was not due to either Fe being present in the starting mineral, simple adsorption of the dye on the catalyst’s surface or direct oxidizing effect of the molecular hydrogen peroxide, without previous catalytic effect. 

## Figures and Tables

**Table 1:** Chemical composition normalized to the content of SiO_2_ in the starting aluminosilicates (*w*/*w* %) and SiO_2_/Al_2_O_3_ mass ratio in raw minerals, starting clays and pillared materials.

Sample	SiO_2_	Al_2_O_3_	Fe_2_O_3_	MgO	TiO_2_	CaO	K_2_O	Na_2_O	SiO_2_/Al_2_O_3_
C1	62.94	17.25	7.86	2.23	0.85	3.00	2.03	3.28	3.65
C2	63.30	19.67	8.17	2.64	0.99	2.19	0.99	1.55	3.22
C3	50.77	17.35	19.94	3.08	1.70	2.61	0.17	3.65	2.93
BV	60.25	23.20	9.37	3.16	1.17	0.93	0.80	0.74	2.60
C1-R	55.09	19.33	13.85	2.87	0.68	1.90	1.13	3.25	2.89
C2-R	60.31	21.82	11.02	2.74	1.09	1.05	1.09	0.54	2.76
C3-R	52.04	17.41	17.96	3.25	1.59	2.33	0.20	3.95	2.99
BV-R	57.57	22.02	10.98	2.60	1.24	1.34	0.90	2.88	2.61
C1-P	54.31	27.52	14.65	2.04	0.67	0.14	0.97	0.35	1.97
C2-P	55.33	30.71	13.39	2.07	1.05	0.09	0.83	0.27	1.80
C3-P	49.53	28.03	19.53	2.60	1.54	0.35	0.14	0.17	1.77
BV-P	53.37	33.26	12.58	2.15	1.11	0.08	0.64	0.19	1.60

**Table 2:** Semi-quantitative mineralogical composition of the clay phases in the raw starting aluminosilicates.

Mineral	Clay Phase	
	Smectite (%)	Illite (%)
BV	60	40
C1	34	66
C2	46	54
C3	54	46

**Table 3:** Iron incorporated, CEC, compensated fraction of CEC (CC), basal spacing and textural properties of raw, refined, and Al/Fe-pillared aluminosilicates.

Sample	Fe_incorporated_^a^ (Fe_2_O_3_ *w*/*w* %)	CEC ^b^ (meq/100 g)	% CC ^c^	*d*_001_^d^ (Å)	S_BET_ (m^2^/g)	S_Ext_ (m^2^/g)	S_µp_ (m^2^/g)
C1	NA	109	NA	12.8	45	30	15
C2	NA	118	NA	15.3	62	47	15
C3	NA	185	NA	12.7	113	75	37
BV	NA	124	NA	14.7	85	49	37
C1-R	NA	149	NA	16.3	105	78	27
C2-R	NA	192	NA	15.0	105	67	38
C3-R	NA	175	NA	12.6	95	67	28
BV-R	NA	137	NA	15.4	97	64	33
C1-P	0.80	82	45	16.9	144	25	119
C2-P	2.37	78	59	17.4	194	29	165
C3-P	1.57	81	54	17.7	185	48	136
BV-P	1.61	91	50	17.6	139	25	114

NA: Not applicable; **^a^** Iron content incorporated in the pillared clays; **^b^** CEC, Cationic Exchange Capacity (dry basis); **^c^** CC, Compensation of the CEC; **^d^** Obtained from powder samples.

## Supplementary Materials

The following are available online at www.mdpi.com/1996-1944/10/12/1364/s1, Figure S1: X-ray diffraction patterns of raw minerals in oriented films: (a) C1, (b) C3 and (c) BV, under following conditions: (1) untreated, (2) Ca^2+^–homoionized, (3) saturated with ethylene glycol and (4) calcined at 400 °C/2 h, Figure S2: Nitrogen adsorption-desorption isotherms of refined and pillared forms of C1 and C3 clays, Figure S3: Hydrogen–temperature programmed reduction (H_2_–TPR) diagrams of refined (R) and pillared forms of clays C1 and BV.
