# Supplementary material for: Preparation of Al/Fe-Pillared Clays: Effect of the Starting Mineral
Source: Materials (Basel). 2017 Nov 28;10(12):1364. doi: 10.3390/ma10121364 (PMC5744299; doi:10.3390/ma10121364)
Supplement: Supplementary file 1 [file materials-10-01364-s001.pdf]

Preparation of Al/Fe-Pillared Clays: Effect of the Starting Mineral

Helir-Joseph Muñoz [1], Carolina Blanco [2], Antonio Gil [3], Miguel-Ángel Vicente [4] and Luis-Alejandro Galeano [1,*]

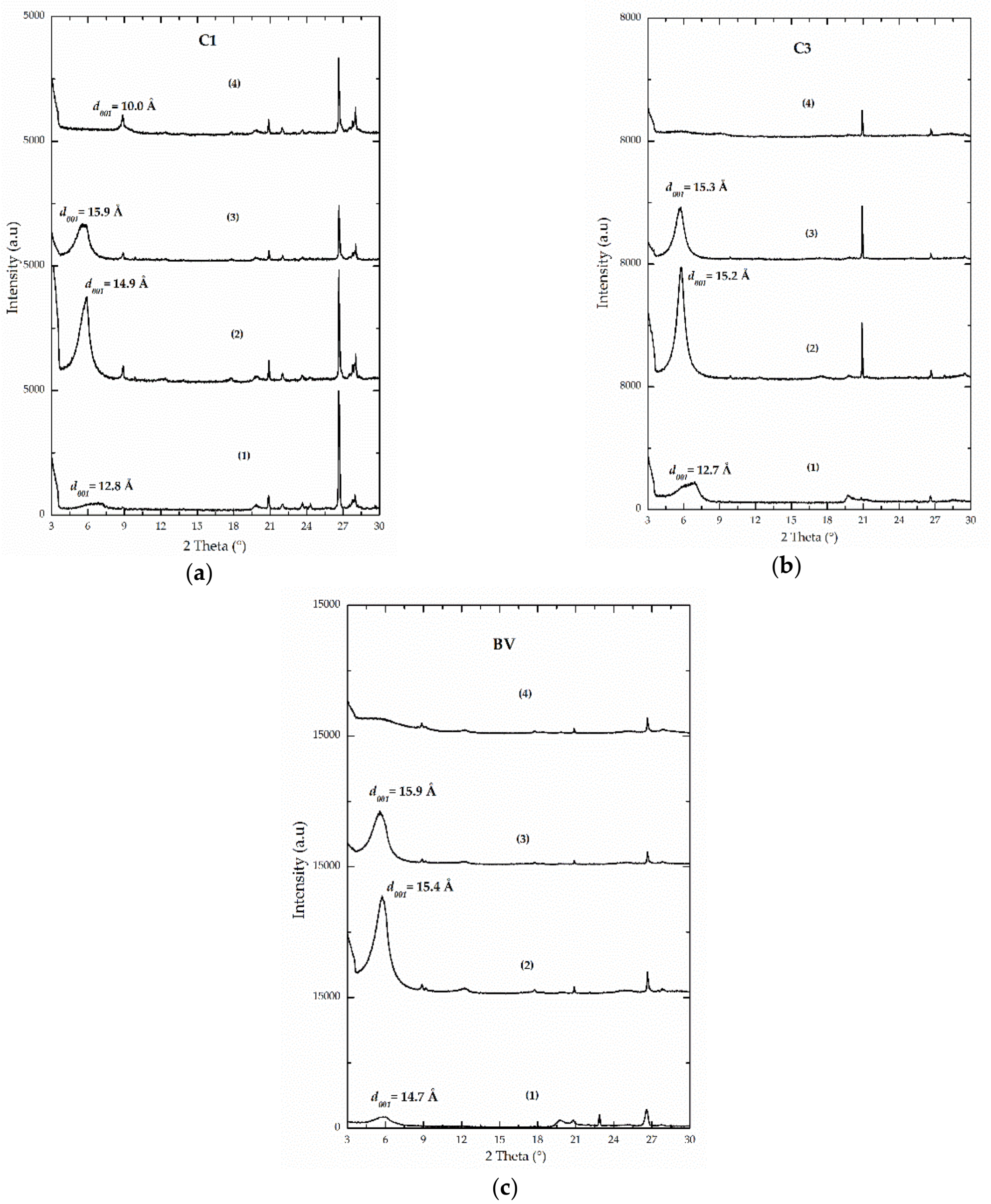

Figure S1. X-ray diffraction patterns of raw minerals in oriented films: (**a**) C1, (**b**) C3 and (**c**) BV, under following conditions: (1) untreated, (2) Ca^{2+}–homoionized, (3) saturated with ethylene glycol and (4) calcined at 400 °C/2 h.

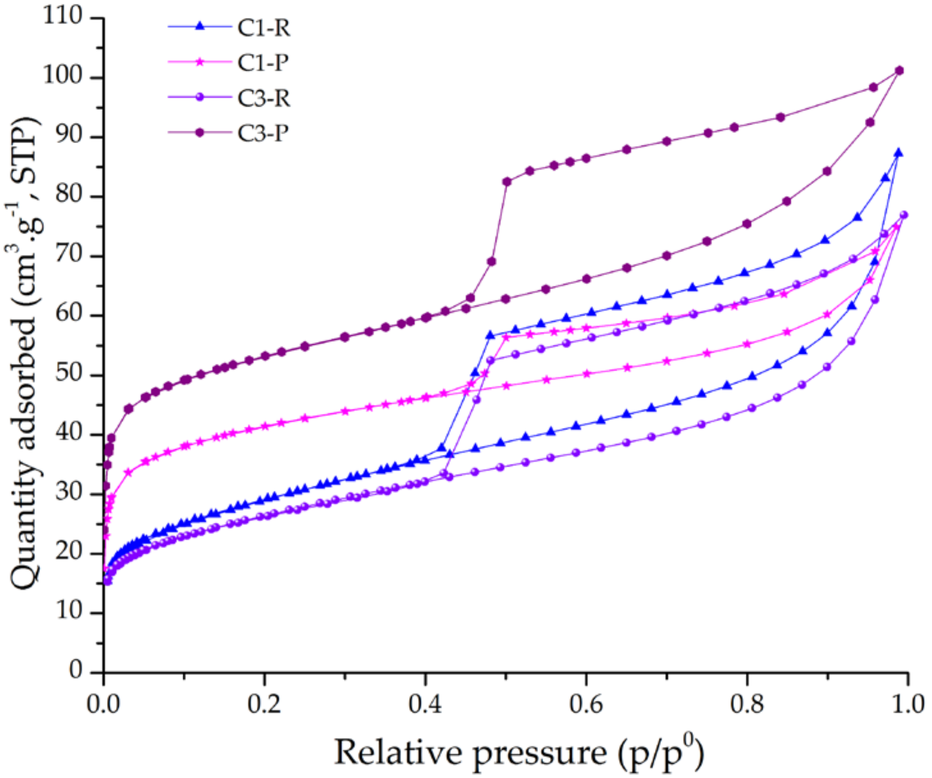

Figure S2. Nitrogen adsorption-desorption isotherms of refined and pillared forms of C1 and C3 clays.

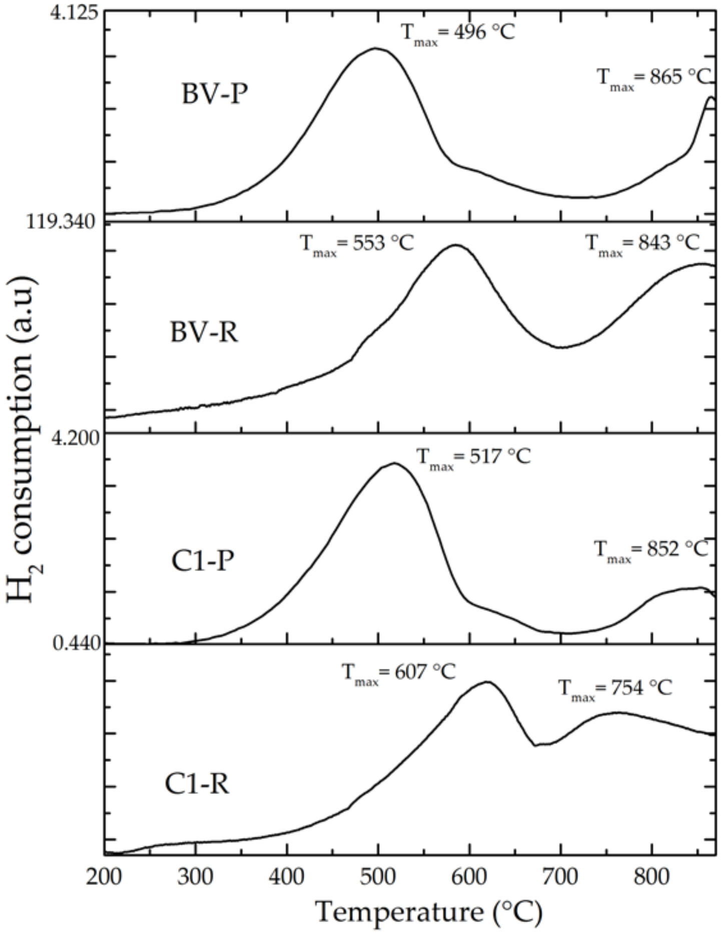

Figure S3. Hydrogen–temperature programmed reduction (H_2–TPR) diagrams of refined (R) and pillared forms of clays C1 and BV.
